# Increased Cell Traction-Induced Prestress in Dynamically Cultured Microtissues

**DOI:** 10.3389/fbioe.2019.00041

**Published:** 2019-03-12

**Authors:** Mathieu A. J. van Kelle, Nilam Khalil, Jasper Foolen, Sandra Loerakker, Carlijn V. C. Bouten

**Affiliations:** ^1^Department of Biomedical Engineering, Eindhoven University of Technology, Eindhoven, Netherlands; ^2^Institute for Complex Molecular Systems, Eindhoven University of Technology, Eindhoven, Netherlands

**Keywords:** prestress, microtissue, *in vitro*, experiments, tissue-engineering

## Abstract

Prestress is a phenomenon present in many cardiovascular tissues and has profound implications on their *in vivo* functionality. For instance, the *in vivo* mechanical properties are altered by the presence of prestress, and prestress also influences tissue growth and remodeling processes. The development of tissue prestress typically originates from complex growth and remodeling phenomena which yet remain to be elucidated. One particularly interesting mechanism in which prestress develops is by active traction forces generated by cells embedded in the tissue by means of their actin stress fibers. In order to understand how these traction forces influence tissue prestress, many have used microfabricated, high-throughput, micrometer scale setups to culture microtissues which actively generate prestress to specially designed cantilevers. By measuring the displacement of these cantilevers, the prestress response to all kinds of perturbations can be monitored. In the present study, such a microfabricated tissue gauge platform was combined with the commercially available Flexcell system to facilitate dynamic cyclic stretching of microtissues. First, the setup was validated to quantify the dynamic microtissue stretch applied during the experiments. Next, the microtissues were subjected to a dynamic loading regime for 24 h. After this interval, the prestress increased to levels over twice as high compared to static controls. The prestress in these tissues was completely abated when a ROCK-inhibitor was added, showing that the development of this prestress can be completely attributed to the cell-generated traction forces. Finally, after switching the microtissues back to static loading conditions, or when removing the ROCK-inhibitor, prestress magnitudes were restored to original values. These findings show that intrinsic cell-generated prestress is a highly controlled parameter, where the actin stress fibers serve as a mechanostat to regulate this prestress. Since almost all cardiovascular tissues are exposed to a dynamic loading regime, these findings have important implications for the mechanical testing of these tissues, or when designing cardiovascular tissue engineering therapies.

## 1. Introduction

Cardiovascular tissues display significant levels of prestress. This prestress is an intrinsic stress which is relieved when the particular tissues are isolated from their *in vivo* environment. The presence of prestress has profound implications for the *in vivo* functioning of cardiovascular tissues. First, prestress directly influences the apparent *in vivo* mechanical properties of, heart valves (Amini et al., 2012; Rausch and Kuhl, 2013) and arteries (Dobrin et al., 1975; Chuong and Fung, 1986; Cardamone et al., 2009), for example. It therefore largely dictates the functioning of these cardiovascular tissues. Second, prestress development has shown to increase tissue extracellular matrix (ECM) alignment and increased matrix deposition in tissue engineered (TE) sheets (Grenier et al., 2005) and heart valves (Mol et al., 2005), respectively, hence influencing structural adaptation in the long run. Finally, abnormal levels of prestress can give rise to serious pathologies which, among others, include vascular hypertension caused by excessive prestress-induced vasoconstriction (Fagan et al., 2004), and aneurysm formation caused by insufficient levels of prestress in tissue-engineered vascular grafts (Tara et al., 2015). In this context, gaining insight into the factors influencing the development of tissue prestress is of paramount importance.

The development of tissue prestress in cardiovascular tissues typically arises due to complex growth and remodeling phenomena, which are only partially understood (Ambrosi et al., 2011). One particularly interesting mechanism for prestress development is the ability of cells to apply traction forces to their surroundings. These forces are generated by contraction of cellular actin stress fibers. Subsequently, these actively generated forces are transferred to the surrounding ECM by means of focal adhesions, leading to the development of tissue prestress. Van Vlimmeren et al. (2012) showed that these cell-mediated traction forces are accountable for roughly 40% of the prestress present in statically cultured tissue-engineered strips.

Many previous studies have investigated the effect of cellular traction forces on the development of tissue prestress. For instance van Loosdregt et al. (2018) studied the relationship between intrinsically generated cell stress and cellular organization in 2D, and found the two to be independent from each other. In addition, Legant et al. (2009) developed a platform in which micrometer-scale cantilevers were used to simultaneously culture 3D microtissues and measure the generated stress. This stress increased with higher cantilever stiffness, but decreased with increasing collagen concentrations. Kural and Billiar (2014) used similar microtissues to study the effect of boundary stiffness, and TGF-β exposure to the developed cell-generated forces. Finally, Boudou et al. (2012) also created a microfabricated platform to measure the dynamic contraction of cardiac microtissues, which was later adapted by van Spreeuwel et al. (2014), who studied the influence of matrix (an)isotropy on this intrinsic contraction. The main advantages of these micrometer scale setups over conventional platforms are the relatively short culture times, and the option of accommodating a large number of samples. However, these particular setups were only used to study cell-generated stress under static external loading which is not physiological for cardiovascular tissues, since these are constantly being exposed to dynamic loading conditions.

There is evidence that external dynamic loading can alter (micro)tissue organization and potentially the degree of developed prestress. Like Legant et al. (2009) and Foolen et al. (2012) also used cantilever-suspended tissues, but in this particular case the cantilevers were mounted on top of a stretchable membrane, enabling dynamic loading of the constructs. They found that uniaxial and biaxial cyclic stretch differentially affected active actin and collagen (re)organization in 3D. However, as the cantilevers were relatively stiff, tissue prestress could not be quantified. A similar study by Gould et al. (2012) found that dynamic loading of collagen hydrogels, in addition to regulating collagen fiber alignment and cellular orientation, is a potent regulator of cellular phenotype. Finally, Zhao et al. (2013) cyclically loaded microtissues for 15 min using electromagnetic tweezers and found increased tissue stiffness due to increased cellular traction forces. However, it remains unclear whether long-term exposure to dynamic mechanical stimuli also induces a cell traction-mediated increase in prestress. This can be especially important in cardiovascular tissue-engineering therapies, which introduce a previously unloaded construct into a continuously dynamic loaded *in vivo* environment. If this is the case, prestress-induced changes in TE construct mechanical properties can alter its *in vivo* functionality, ultimately determining the success or failure of the therapy.

Delvoye et al. (1991) showed that in constrained collagen gels, seeded fibroblasts will compact the ECM until the tensile stress reaches a steady state. After subsequent perturbations in the gel, the cells will again strive to restore the same mechanical steady state. In analogy with this phenomenon, we hypothesize that cells will also mediate tissue prestress in response to dynamic stimulation by increasing their actin-generated cell traction forces.

To investigate the validity of this hypothesis, in this study 3D microtissues were exposed to long-term dynamical loading, after which the cell traction-induced prestress was quantified. To this end, a microfabricated tissue gauge (μTUG) platform (van Spreeuwel et al., 2014) was combined with the commercial available Flexcell system to create a μFlex-TUG setup and facilitate 24 h cyclic stretching of the microtissues. First, the setup was validated by measuring the microtissue stretches during dynamic culture using digital image correlation. Subsequently, microtissues were dynamically cultured for 24 h, which increased the cell traction-induced tissue prestress almost two-fold. Next, the origin of the increased prestress levels was investigated. First, a ROCK-inhibitor, temporary inhibiting stress fiber contractility, was added after 48 h to both dynamically and statically cultured experimental groups. In both groups, prestress levels were comparable and almost completely abated after ROCK-inhibition, showing that the elevated prestress levels after 24 h dynamical culture can solely be attributed to increased stress fiber contraction. Second, after subsequent removal of the ROCK-inhibitor, prestress magnitudes returned to static control levels. In addition, in another dynamically cultured group, the elevated prestress levels returned to magnitudes comparable to static controls after removal of the dynamic cue. These findings show that intrinsic tissue prestress is a highly regulated parameter, in which the actin stress fibers serve as a mechanostat to control this prestress.

Since cardiovascular tissues are experiencing everlasting hemodynamic loading, and the fact that prestress influences a tissue's mechanical behavior, these findings have important implications for accurately determining (*in vivo*) mechanical properties. Additionally, tissue-engineering therapies aimed at replacing such cardiovascular tissues often use cells with a contractile phenotype. The findings suggest that dynamic stimulation after implantation of the TE constructs could alter their *in vivo* function and subsequent success of the therapy.

## 2. Methods

### 2.1. μFlex-TUG and μTUG Fabrication

**Figure 1:**
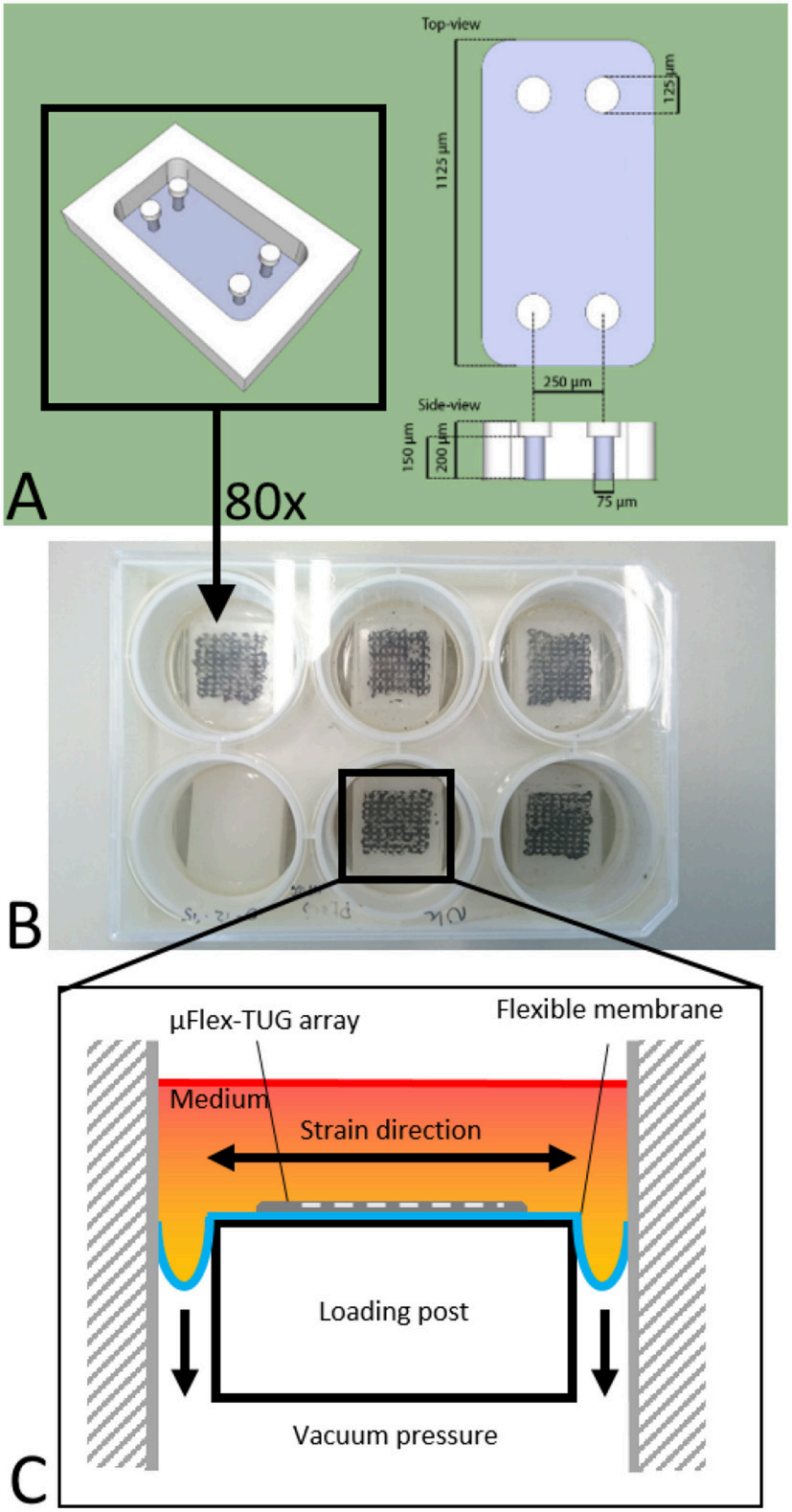
**(A)** Design of the microwell containing four PDMS microposts (courtesy of Alex Bastiaens). **(B)** μTUGs created on a Flexcell plate to create a μFlex-TUG. **(C)** Application of uniaxial dynamic loading to the μFlex-TUGs.

The setup consists of eighty microfabricated tissue gauges (μTUGs), where each of these μTUGs contains four compliant polydimethylsiloxane (PDMS) microposts embedded in a microwell (Figures 1A,B). Fabrication of the μTUGs was performed according to van Spreeuwel et al. (2014). Briefly, positive masters were created by spincoating SU-8 photoresist (Microchem, Berlin, Germany) on a silicon wafer, which was subsequently UV-exposed. Alignment of different layers was performed using a Suss MJB3 mask aligner (Suss Microtec, Garching, Germany). The masters were then made non-adhesive through overnight silanization with (tridecafluoro-1,1,2,2-tetrahydrooctyl)-1-trichlorosilane (Abcr, Karlsruhe, Germany) under vacuum. Negative PDMS templates were made by casting PDMS, with prepolymer to curing agent ratio of 10 : 1 w/w (Sylgard 184; Dow-Corning, Midland, USA) on the masters, followed by overnight incubation at 65°C. These negative PDMS templates were then treated in a plasma oxydizer (1 min at 100 watt) and again made non-adhesive by overnight silanization. Subsequently, PDMS was cast on these templates which was then degassed in a vacuum oven. The PDMS-covered negative templates were either stamped in Flexcell BioFlex plates (Flexcell International Corporation, Burlington, NC, USA) to create μFlex-TUGs for dynamic culture, or in regular petri dishes to create static μTUGs. Finally, both setups were cured overnight at 65°C followed by careful removal of the negative template.

### 2.2. Cell and Microtissue Culture

**Figure 2:**
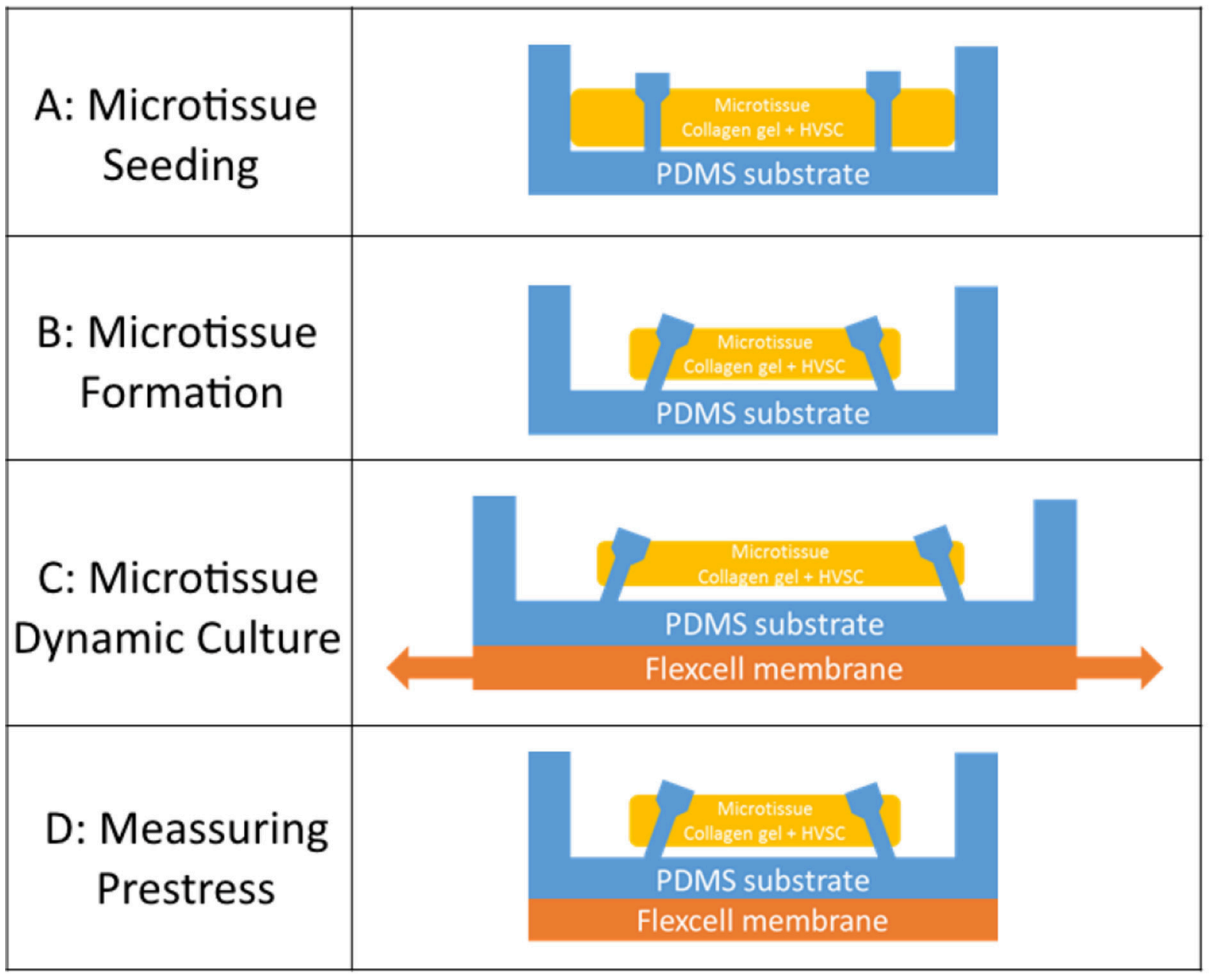
**(A)** Seeding of the cell/gel suspension in a microwell. **(B)** Formation of a microtissue. **(C)** Dynamic stretching of a microtissue. **(D)** Measuring prestress by tracking the micropost displacement.

Human vena saphena cells (HVSCs) were cultured until passage 7 using culture medium containing advanced Dulbecco's Modified Eagle Medium (DMEM, Invitrogen, Carlsbac, CA, USA), supplemented with 10% Fetal Bovine Serum (FBS, Greiner Bio One, Frinckenhausen, Germany), 1% Glutamax (Invitrogen) and 1% penicillin/streptomycin (Lonza, Basel, Switzerland). These cells have previously been characterized as myofibroblasts (Mol et al., 2006) and exhibit a contractile phenotype. Microtissues were created according to the protocol described by van Spreeuwel et al. (2014). In short, first the μFlex-TUGs and μTUGs were sterilized by immersion in 70% ethanol for 15 min, followed by 15 min UV radiation. To impair cell adhesion, the PDMS was treated with 0.2% Pluronic F127 (BASF, Ludwigshafen am Rhein, Germany) in PBS for 15 min. A gel mixture containing 50% collagen (Rat tail collagen type 1, BD biosciences, New Jersey, US, 3.2 mg ml^−1^), 39% culture medium, 8% growth factor-reduced Matrigel (BD Biosciences) and 3% 0.25 M NaOH was prepared and centrifuged into the microwells (1 min, 2,000 RPM). Residual gel which was not in the microwells was used to resuspend harvested HVSCs, after which this suspension was centrifuged again into the microwells (1 min, 1000 RPM) (Figure 2A). Excessive gel was removed and the remaining cell/gel suspension in the microwells was allowed to polymerize for 10 min at 37°C. Finally, 0.25 mg/mL L-ascorbic 2-phosphate acid (Sigma-Aldrich, St. Louis, MO, USA) supplemented standard culture medium was added on top of the microwells. After seeding, the setups were placed in an incubator for 24 h at 37°C, 100% humidity and 5% CO_2_ to allow for initial microtissue formation (Figure 2B).

### 2.3. Validation of Microtissue Strain in μFlex-TUG

To cyclically stretch the microtissues, the seeded μFlex-TUGs were placed in the Flexcell FX-4000 system supported by rectangular posts (Figure 1B). This system enables application of uniaxial dynamic loading conditions by applying a vacuum to the flexible membrane of the Flexcell plates and stretching it over the posts (Figure 1C). The PDMS microposts connected to the membrane translate these stretches to the connected microtissue (Figure 2C). It is unknown how the Flexcell membrane stretches translate to actual microtissue stretches. In this regard, Colombo et al. (2008) showed that accurate calibration and measurements of Flexcell strains are recommended given the viscoelastic nature of the Flexcell system. Therefore, the strains in the microtissues in the μFlex-TUG were validated by means of digital image correlation (DIC). Toward this end, microtissues were seeded in the μFlex-TUG system and 5,10,15 and 20% Flexcell strains with a frequency of 0.5 Hertz were applied. Videos of the stretched microtissues were recorded with a camera mounted on a Zeiss stereo discovery v8 (Oberkochen, Germany) and analyzed using previously developed DIC software by Neggers et al. (2012) to obtain the Green-Lagrange strains (in the constrained tissue direction) in one rectangular middle section of the microtissue for each of the applied Flexcell strains.

### 2.4. Confocal Microscopy

To visualize microtissue structure at the end of the experiments, the microtissues were incubated with a collagen-specific CNA35 probe (Boerboom et al., 2007) for 30 min, after which Z-stack images were made using a confocal laser scanning microscope (TCS SP5X; Leica Microsystems, Wetzlar, Germany, excitation 488 nm, emission 520 nm, magnification 10x, Z-step= 3 μm). Next, microtissues were fixed for 10 min using 10% formalin, followed by permeabilization with 0.5% Triton X-100 in PBS. The cell nuclei and actin network were stained with DAPI and Atto 488 Phalloidin (Sigma-Aldrich) dyes, respectively, and imaged using the confocal microscope.

### 2.5. Prestress Measurements

In order to quantify the tissue prestress (Figure 2D), first the generated forces on the four PDMS microposts were determined. Toward this end brightfield images of the microtissues were made on every time point on an EVOS XL Core microscope (Thermo Fisher, Waltham, MA, USA). Using a semi-automatic, custom-made Matlab script (Mathworks, Natick, USA), the four strongest circles in each image were detected using Matlab's *imfindcircles* function. The position of these circles corresponded to the top of the four microposts. The displacement of the center of each circle with respect to its original position was used to determine the displacement of the top of these posts (*u*). Next, the spring constant *K* [= 1.22 N m^−1^] of a single micropost was determined by means of finite element simulations (Abaqus, Dassault Systèmes Simulia Corp., Providence, RI, USA, version 6.14-1). First, a force was applied to the part of a single micropost where the tissue is attached, which is just below the relatively large cap of the post. Next, the displacement of the top middle node in the direction of the force was determined (for more information see the Supplementary Material). Second, using this spring constant and the micropost displacement, the force exerted on one micropost ($F_{post}^{i}$) was determined:

(1) $$\begin{array}{r} F_{post}^{i}=Ku_{post}\;and\;F_{tissue}=\frac{\sum_{n=1}^{4}F_{post}^{i}}{2} \end{array}$$

**Figure 3:**
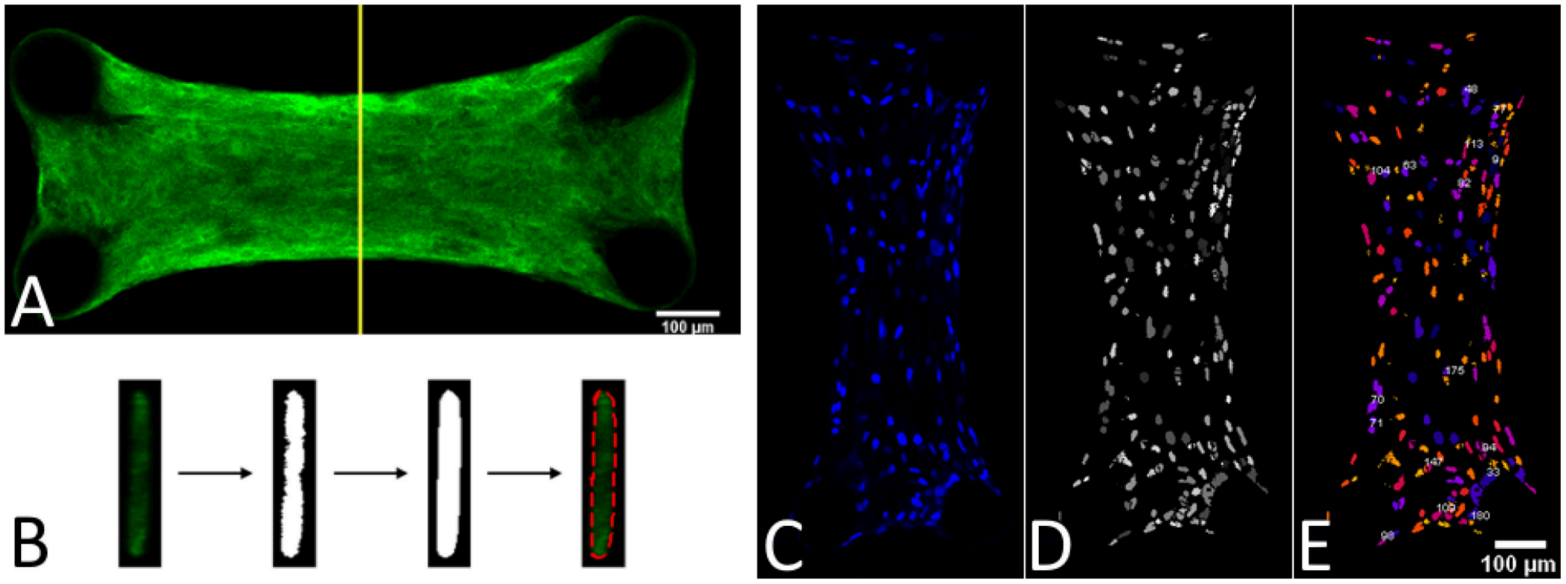
**(A)** Middle slice of a 3D image form a collagen-stained microtissue. **(B)** Determining the cross-sectional area by using a convex hull on the middle lateral slice form the 3D image. **(C)** DAPI stained a microtissue. **(D)** Watershed filtering of cell nuclei. **(E)** Segmenting and counting the cell nuclei in 3D, where every individual nucleus has a different color.

**Figure 4:**
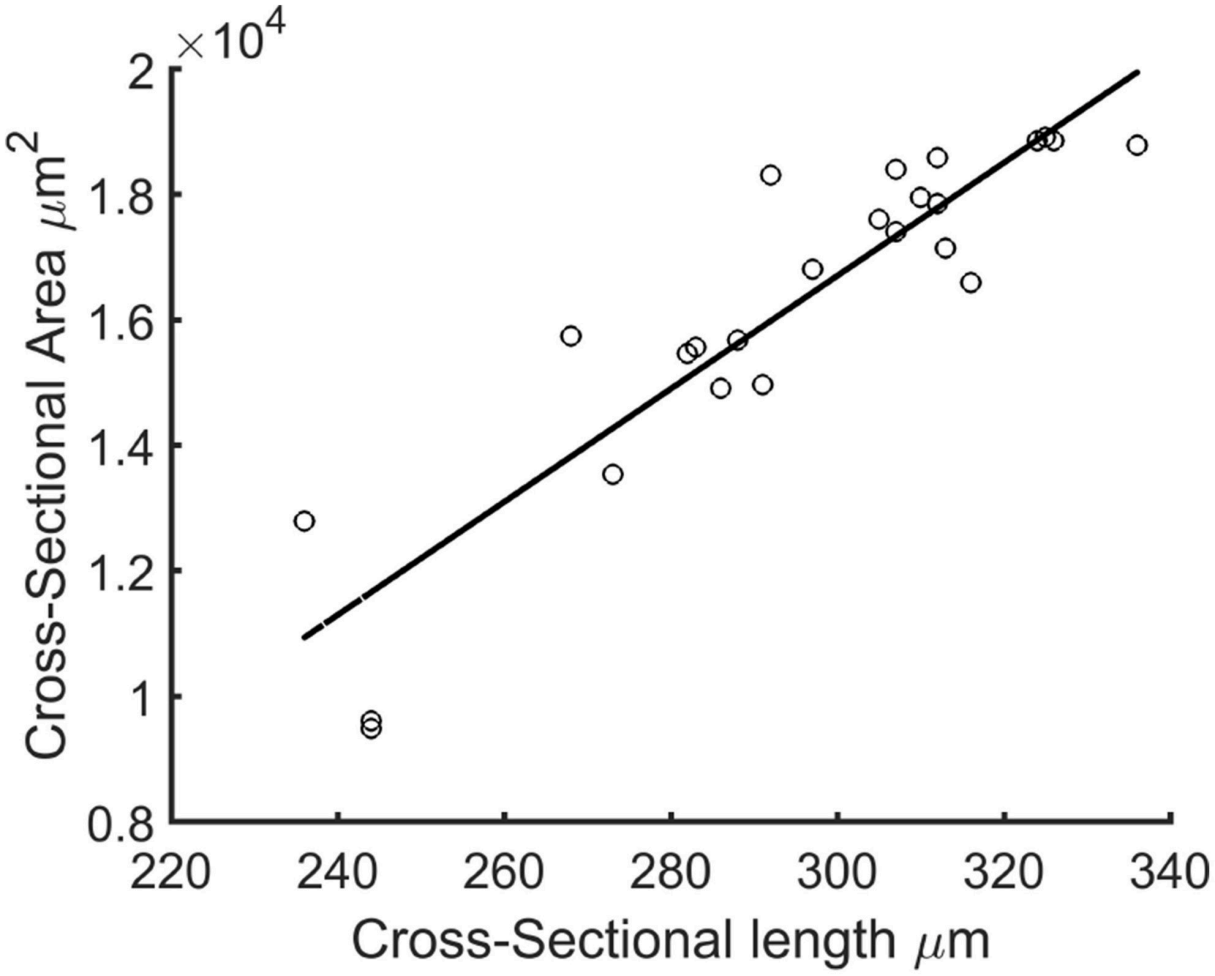
Cross-sectional length (*A*_*CSL*_) plotted against actual microtissue CSA *A*_*CSA*_. The black line is the fitted linear model with equation: *A*_*CSA*_=90.09^*^A_*CSL*_-10323.41 (*R*^2^=0.91).

Since the force generated by the microtissue is equal and opposite on the microposts on both side of the tissue, the total microtissue force *F*_*tissue*_ is the sum of the individual forces on each of the four posts divided by two. It needs to be noted that only the component of the total force in the constrained direction was considered. To acquire the prestress in the microtissues, the measured forces need to be translated into stresses. Toward this end, the cross-sectional area (CSA) from each microtissue was obtained. First from all the images made during the force measurements, Matlab's *imdistline* function was used to obtain the width in the middle part of the microtissue, which we define as the “Cross-Sectional Length” (*A*_*CSL*_). Second, at the end of the experiment the “real” CSA (*A*_*CSA*_) from the collagen stained microtissues (section Confocal Microscopy) was determined. All slices of the Z-stack were imported into Matlab, and from the middle image the main orientation of the largest connected component (which is the microtissues) was obtained using the *regionprops* Matlab function. This main orientation was used to rotate all individual slices so that the images align in the horizontal direction (Figure 3A). Next, each lateral slice form the 3D image was binarized. A convex hull was wrapped around the images, where the pixels within this convex hull compose the CSA (Figure 3B). The lateral slice with the smallest convex hull was obtained and used together with the dimension data from each voxel to obtain the microtissue CSA in μm^2^. This real CSA was plotted against the microtissue width at the end of the experiment, for which a strong correlation (*R*^2^=0.91) was found when fitting a linear model (Figure 4). Finally, the real CSA was determined by applying the obtained linear model to the measured microtissues widths at each previous time point of the experiment. All the forces (*F*_*tissue*_, μN) were divided by the CSA (*A*_*CSA*_, μm^2^) to obtain the microtissue prestress (σ) in kPa, i.e.,

(2) $$\begin{array}{r} \sigma =\frac{F_{tissue}}{A_{CSA}}. \end{array}$$

### 2.6. Number of Cells

To determine the prestress magnitude per cell, the number of cells per microtissue was counted. This was done by importing the Z-stacks of the DAPI channel from the confocal images into ImageJ (NIH, Bethesda, MD, USA) (Figure 3C). First the stacks were filtered with a 3D watershed algorithm (Ollion et al., 2013) (Figure 3D). Next the amount of cells in the binarized images were counted using a 3D object counter plugin (Bolte and Cordelières, 2006) (Figure 3E).

### 2.7. Experimental Design

**Figure 5:**
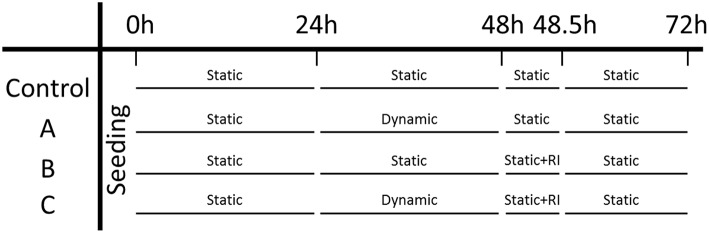
Experiment design: the left column indicates the three experimental groups and the control group. Each time point is indicated on the top row.

Microtissues were seeded in two μTUGs as a static control, and additionally two wells in a μFlex-TUG were seeded as a dynamic group. An overview of the experimental design can be found in Figure 5. Initially, all four groups were cultured under static conditions for 24 h to allow the formation of microtissues, after which the force and CSA were determined. To determine the effect of dynamic loading on the generated prestress, subsequently the two dynamic groups were switched to dynamic culture conditions by applying 10% strain to the Flexcell plate at 0.5 Hz (Foolen et al., 2012). After another 24 h, the force and CSA was determined again for all groups. To analyse the cell traction-mediated fraction of the total prestress, a ROCK-inhibitor (Y-27632, Sigma-Aldrich) was added to one static and one dynamic group and forces and CSA were measured again after 30 min. Finally, to determine whether differences in prestress were reversible, the ROCK-inhibitor was removed by adding fresh culture medium and switching all dynamic groups back to static culture conditions. After a final 24 h the prestress was measured again for all groups.

### 2.8. Statistical Analysis

Only microtissues which were still attached to all four posts at the end of the experiment (72h) were included in the analysis. For the static control group and the ROCK-inhibited static group, sample numbers were 21 and 24, respectively, while for the dynamic and ROCK-inhibited dynamic group the sample sizes were 10 and 13, respectively. All data were reported as mean ± standard error of mean. Statistical analysis of the data was performed with a many-to-one Dunnett test, comparing all conditions to one control group, accounting for heterogeneous variances and unequal samples sizes using the methods and implementation into the statistical software package R (R Core Team, Vienna, Austria) described in Herberich et al. (2010). For analyzing the number of cells per microtissue, a Wilcoxon signed-rank statistical test was performed in MATLAB.

## 3. Results

### 3.1. Microtissue Strains Are One-Third of the Applied Flexcell Strains

**Figure 6:**
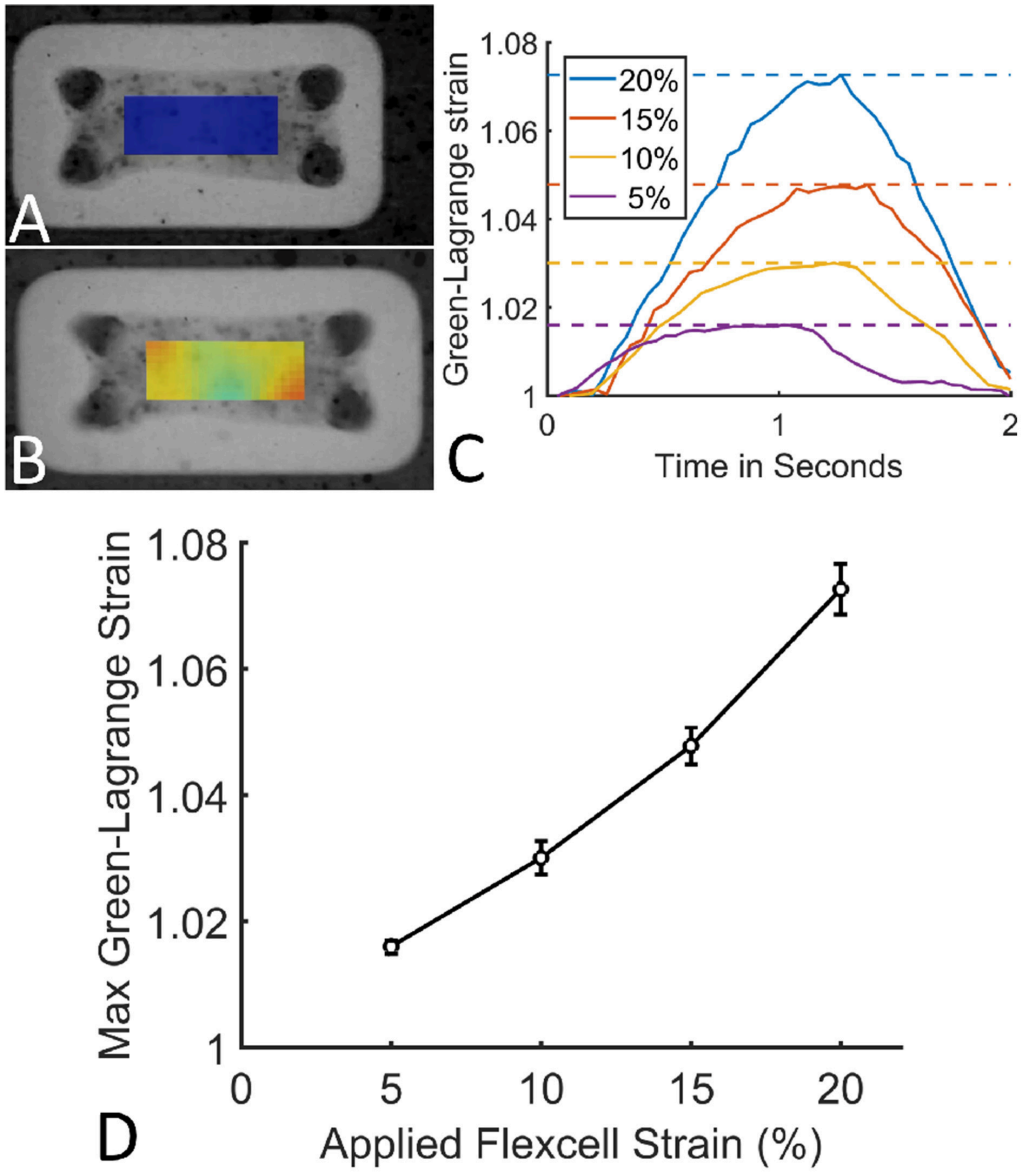
**(A)** A microtissue without externally applied mechanical cues. **(B)** A dynamically cultured microtissue at 10% Flexcell strain. **(C)** Average actual microtissue Green-Lagrange strains during one cycle (2 s) for different applied Flexcell strains (legend). The dotted lines indicate the maximal strains during the cycles. **(D)** Maximum Green-Lagrange strains (averaged over the mid-section of the microtissues in **B**) for increasing applied Flexcell strains.

The applied Flexcell strains clearly translated to a strain in the microtissues (Figures 6A,B). During one cycle (2 s), these microtissues strains followed a homogeneous inverse parabolic profile (Figure 6C), reaching a maximum value halfway through the cycle. When quantifying the maximal microtissue Green-Lagrange strains, it was found that they increased with the applied Flexcell strains (Figures 6C,D). However, the applied Flexcell strains did not equal the microtissue strains. As a rule of thumb, the actual microtissue strain was assessed to be one-third of the applied Flexcell strain.

### 3.2. The Microtissues Have a Uniform Distribution of Cells, and Collagen and Stress Fibers Are Aligned in the Constrained Direction

**Figure 7:**
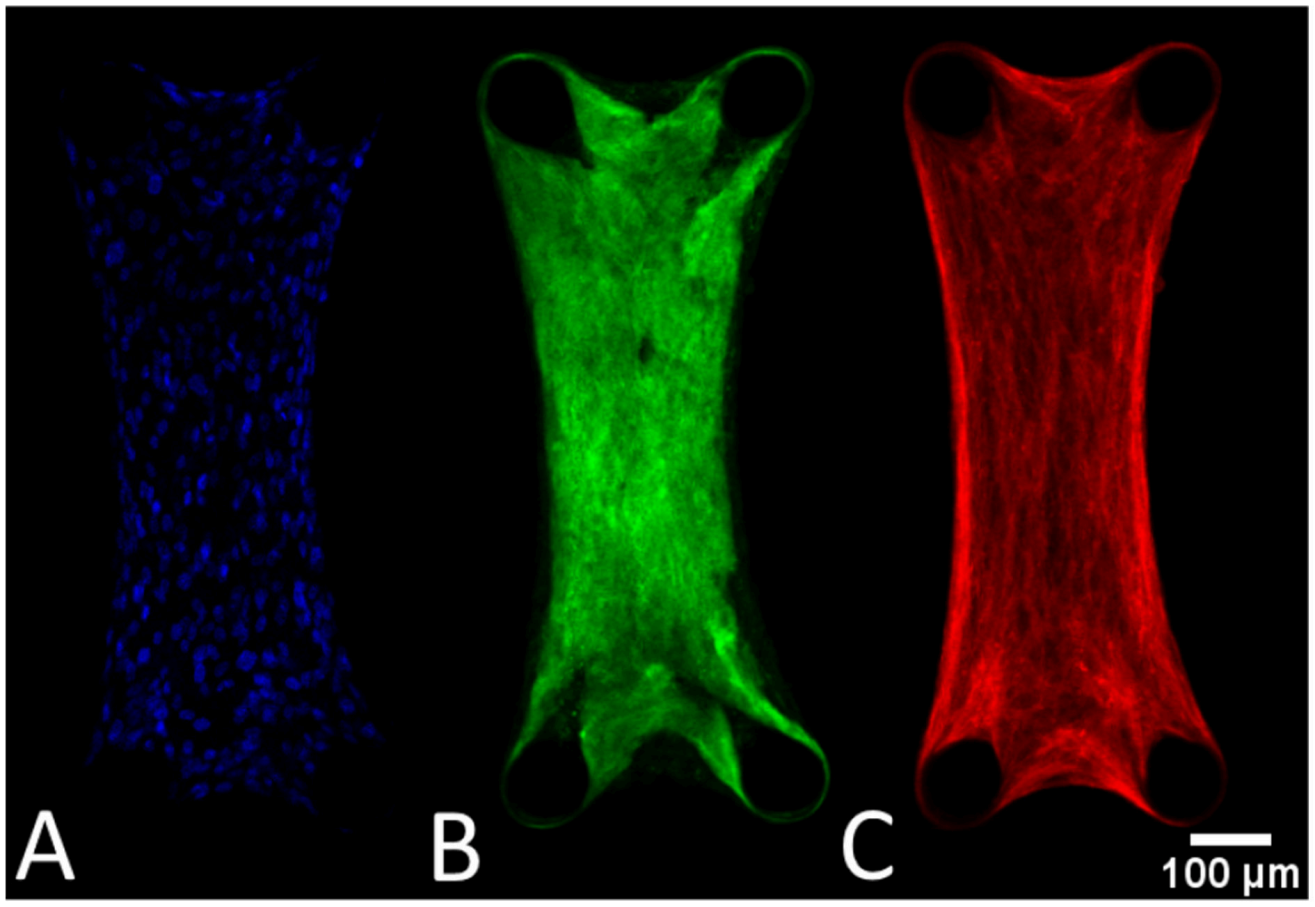
General tissue organization depicted in these confocal images of a DAPI **(A)**, collagen **(B)**, and actin-stained **(C)** microtissue after 72 h of culture.

The cells are uniformly spaced inside the microtissues (Figure 7A). Also the collagen (Figure 7B) and actin (Figure 7C) are homogenously distributed in the microtissue, where the fibers are oriented in the longitudinal direction.

### 3.3. Increased Prestress in Dynamically Cultured Microtissues

**Figure 8:**
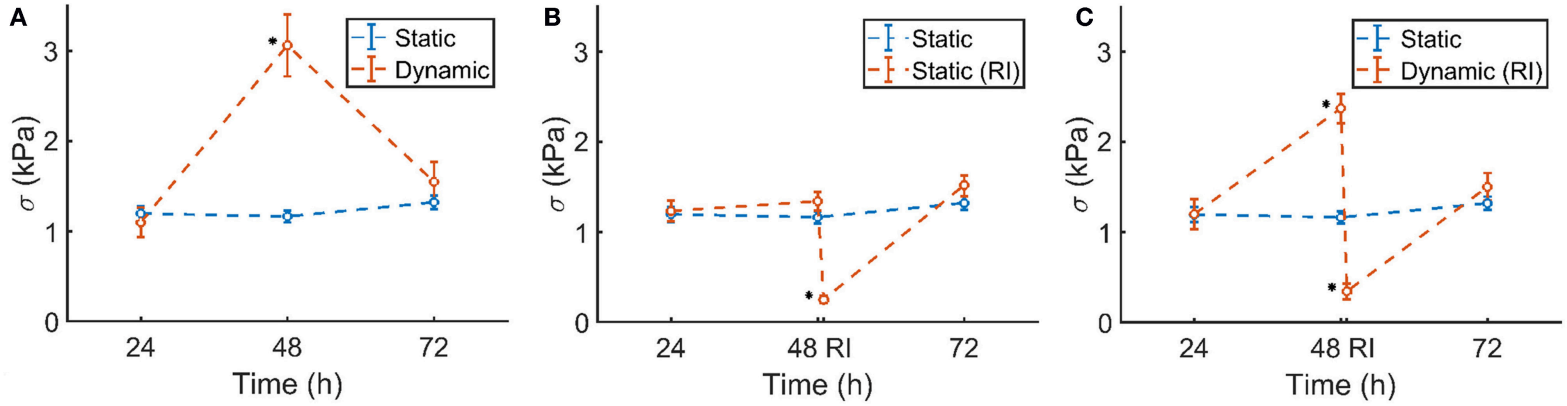
**(A)** Static control group (blue) and dynamically cultured microtissues (red). **(B)** Static control group (blue) and ROCK-inhibited (RI, red, at 48 h) microtissues over the course of 72 h. **(C)** Static control group (blue) and dynamically stretched microtissues (red). A ROCK-inhibitor was added to the dynamically cultured microtissues after 48 h (RI), which was again removed after 30 min. Significant changes (*p* <0.05) between the two groups are indicated by a ^*^. Note that the blue controls in **(A–C)** are the same experimental group in all figures. The characters of subfigures **(A–C)** correspond to the characters used in Figure 5.

The prestress in the static control group remained constant during 72 h of culture (Figure 8A, blue). Although initially the prestress was similar after 24 h, following dynamic stimulation, the microtissue prestress increased significantly to levels over twice that of the statically cultured controls after 48 h of culture (Figure 8A, red). Upon removal of the dynamic mechanical cue, prestress levels went back to equal magnitudes as that of static controls at 72 h of culture.

### 3.4. Prestress Increase Is Caused by the Stress Fibers and Is Reversible

The statically cultured microtissues which were exposed to the ROCK-inhibitor showed a similar prestress magnitude at 24 and 48 h of culture. However, after addition of the inhibitor, the prestress dropped significantly (Figure 8B, red), leaving only a small amount of residual stress. After subsequent removal of the ROCK-inhibitor, within 24 h the prestress again reached comparable levels compared to the control group.

A similar phenomenon was observed in the ROCK-inhibited dynamically cultured microtissues. Upon dynamic culture, the stretched microtissue prestress again increased significantly to levels over twice that of the statically cultured controls at 48 h of culture (Figure 8C, blue). Addition of the ROCK-inhibitor diminished that higher prestress almost completely, leading to levels of residual stresses comparable to the statically ROCK-inhibited microtissues (Figure 8B). In line with earlier observations, removal of the ROCK-inhibitor and subsequent static culture resulted in similar prestress levels as statically cultured controls after 72 h.

### 3.5. Prestress Per Cell Is Similar in Statically and Dynamically Cultured Groups at the End of Culture

**Figure 9:**
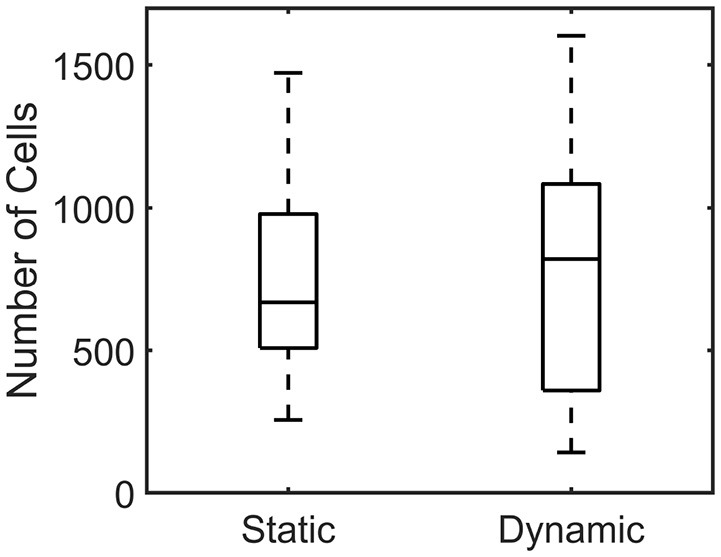
Boxplot of the number of cells per microtissue for the statically **(Left)** and dynamically **(Right)** cultured groups at the end of the experiment.

The number of cells per individual microtissue was determined using a DAPI staining (section Number of cells) at the end of culture. The number of cells differed substantially within the experimental groups for both conditions (Figure 9). However no statistical differences between the statically and dynamically cultured groups were found (*p* = 0.5614).

**Figure 10:**
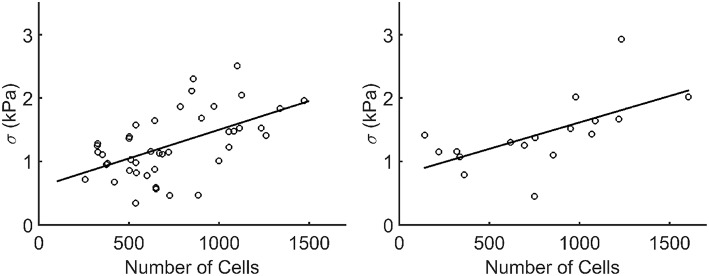
Stress plotted against the number of cells (*N*_*cells*_) per microtissue for the statically (left) and dynamically (right) cultured groups at 72 h of culture (after recovery). Each dot corresponds to one microtissue. The black lines indicate a linear fit through all data points. The equations for these lines are σ = 0.0009^*^N_*cells*_+0.5957 and σ = 0.00085^*^N_*cells*_+0.7825 for the statically (*R*^2^=0.55) and dynamically (*R*^2^=0.62) cultured microtissues, respectively.

When plotting the stress in each microtissue against the number of cells, an increasing linear relationship was found for both the statically (*R*^2^=0.55) and dynamically (*R*^2^=0.62) cultured groups (Figure 10).

## 4. Discussion

In this study it was investigated how long-term exposure to dynamic loading will influence cell traction-induced prestress in 3D microtissues. To facilitate this, a microfabricated tissue gauge platform was combined with the commercially available Flexcell system to enable 24 h cyclic stretching of the microtissues.

### 4.1. Setup Validation

First the developed system was validated by measuring the actual microtissue strains with DIC. As already stated by Colombo et al. (2008), the applied Flexcell strains do not necessarily translate one-to-one to actual microtissue strains. On average, the actual microtissue strain was one third of the applied Flexcell strains. Possible reasons for this are two-fold: first, addition of a PDMS layer to the Flexcell membrane makes the entire base of the μFlex-TUGs more stiff, and thus with the same vacuum magnitude the membrane yields less displacement. Second, the microtissues are connected to compliant microposts, which contrary to for example Foolen et al. (2012), bend when the cyclic stretch is applied, making the strain in the microtissues even smaller.

### 4.2. Cell Traction Forces Are a Mechanostat for Tissue Prestress

After validation of the setup, experiments were performed to determine the effect of dynamic loading on cell traction-mediated prestress. In the first 24 h, all groups were cultured statically to ensure microtissue formation. The prestress after 24 h was similar for all four groups. This confirms that merely culturing the microtissues in a different setup (μFlex-TUG or static μTUGs) without applying cyclic stretch did not affect the prestress. For static controls, prestress remains constant over the entire experiment. Upon dynamic culture, the prestress roughly increased two-fold compared to the static controls. This prestress increase is entirely caused by cell traction forces, as ROCK-inhibition almost completely diminishes the prestress. In fact, both the statically and dynamically cultured microtissues returned to similar values of prestress (0.25 and 0.34 kPa, respectively) after ROCK-inhibition. This passive residual stress in the static group contributes 19% to the total prestress, and 14% in the dynamic group. When comparing the percentual contribution in the static group to statically cultured TE strips by Van Vlimmeren et al. (2012), this percentage is significantly lower (60%). However, in this particular study the culture times were considerably shorter, so very little matrix production could have taken place. Therefore, this discrepancy could be explained by the fact that the only residual matrix prestress is caused by the relatively compliant collagen gel, which is far less stiff compared to the fibrous collagenous matrix in TE strips.

After switching the dynamically cultured microtissues back to static conditions, or upon removal of the ROCK-inhibitor, prestress in all groups is again normalized and comparable to the control group after 72 h. The typical timescales (smaller than 24 h) at which the increase and decrease of prestress lay within the typical turnover rate of actin stress fiber, which is approximately minutes (Peterson et al., 2004; Mbikou et al., 2006; Livne and Geiger, 2016). These results show that cyclic load-induced elevated prestress levels, and the restoration to baseline levels after removal of the ROCK-inhibitor or dynamic cue are regulated only by the actin stress fiber generated cell traction forces. It appears that in these relatively immature microtissues, the actin stress fibers serve as a mechanostat to regulate the prestress in response to perturbations in the environment.

### 4.3. Microtissue Structure and Increasing Prestress With Cell Number

At the experimental timepoints, both statically and dynamically cultured microtissues were stained for actin stress fibers, collagen and cell nuclei. Figures 7B,C show that the collagen, and cellular actin fibers are present uninterruptedly between the four microposts. This means that all cell-generated forces can be translated within the tissue. Both the actin and collagen fibers are aligned in the longitudinal direction of the microtissues. This phenomenon is not surprising since this is the only constrained direction of the constructs.

The DAPI-stained microtissues affirm a homogeneous distribution of cells with the microtissue (Figure 7A). However, cell numbers among microtissues varied up to 10-fold (± 150–1,500). This is possibly caused by the fact that during seeding, the cells are centrifuged into 80 different microwells at once. This makes it impossible to control how many cells end up per microtissue. Regardless of this fact, successful microtissues formation—attached to all four posts—could occur with such a varying number of cells per construct. The effect of the varying numbers of cells per microtissue is also noticeable in microtissue prestress. Since prestress differences in this study are mainly caused by cell traction forces, larger cell numbers in microtissues cause higher prestress magnitudes. However, a two-fold increase in cell number for instance does not yield a prestress of the double magnitude. It appears that the cells cooperatively regulate the tissue prestress in response to altering cell numbers. This coincides with the findings by Canovi et al. (2016), who showed that cellular traction forces in 2D culture decrease with increasing confluency. Again in this case, cellular traction forces serve as a mechanostat for the total tissue prestress in response to changing numbers of cells.

### 4.4. Challenges and Future Directions

In these experiments, the microtissues were loaded uniaxially. Since many cardiovascular tissues are loaded in multiple directions, a biaxial loading regime as used in Foolen et al. (2012) would mimic the *in vivo* situation more closely. Moreover, this setup could enable testing prestress development in anisotropically organized tissues. In addition, currently it was not investigated how the dynamic loading might affect cellular morphology and phenotype. Regardless of the relatively short time of the experiments, such morphological and phenotypic changes are known to occur, for instance under the influence of micropost stiffness Kural and Billiar (2016). With this platform, a thorough investigation of morphology and/or phenotype could be conducted using this platform. Moreover, although the large range of cells per tissue can at first be perceived as a drawback of the current method, it could be utilized to investigate the effect of cell number on generated tissue force and prestress more thoroughly. For instance, a comprehensive analysis of the force or stress per cell could help to determine the cooperative behavior of cells to reach a tensional homeostasis. Also, in the current experiments the applied microtissue strains were relatively low (3.01% ± 0.26), whereas higher levels of strain would be more realistic to the native situation of many cardiovascular tissues. Additionally, tracking prestress levels in real-time, instead of at discrete time points can give more insight into the rate of change in prestress. Currently, it is unknown how prestress changes in between the discrete time points (hence the dotted lines in Figure 8). Finally, the microtissues were created using a collagen gel, supplemented with matrigel. Such collagenous gels are subject to significant degradation and remodeling, which can alter the mechanical properties, and therefore the cellular response (Allison et al., 2009; Smithmyer et al., 2014). However, since collagen gels tend to be stable for culture times shorter than 1 week (Smithmyer et al., 2014), the degree of degradation is probably negligible in the current experiments, which only last 72 h. In that regard, little to no matrix production could occur during these experiments, which explains the relatively small contribution of the residual stress to the total tissue prestress. Yet, it is known that prolonged dynamic loading induces elevated levels of ECM production and cross-linking (Boerboom et al., 2008; van Geemen et al., 2013), which is expected to generate more prestress. It would therefore be interesting to increase the culture time of the experiments to study the effect of ECM-induced prestress.

### 4.5. Implications

The implications of increased cell traction-induced prestress under dynamic loading conditions and subsequent relatively quick recovery, are numerous. This phenomenon should for instance be considered when mechanically testing cardiovascular tissues. Rausch and Kuhl (2013) already reported that neglecting tissue prestress explained differences in reported stiffness values in literature of up to four orders of magnitude for the same types of tissues, showing the importance of accurate prestress quantification. Usually, these prestress measurements involve isolating the tissue in question from its native situation and determining the retraction (Chuong and Fung, 1986; Amini et al., 2012; Horny et al., 2013). However since this study shows that the prestress drops relatively quickly after removal of the dynamic cues, prestress measurements in these dynamically loaded cardiovascular tissues could yield different outcomes depending on the time of the measurement after excision.

Increased cell-induced prestress could also influence tissue-engineering therapies. Since these therapies rely on cell-seeded scaffolds where these cells often have a contractile phenotype (Mol et al., 2006; Tara et al., 2015), substantial prestress magnitudes are ought to be expected. Introducing such a construct from a previously unloaded environment into a continuously dynamic loaded *in vivo* environment, can lead to prestress-induced changes in the construct's mechanical behavior. This can lead to subsequent alterations in its *in vivo* functionality, determining the success or failure of the therapy.

Several factors associated with tissue-engineering strategies can influence these cell-generated prestress levels. For instance, the mechanical properties of the scaffold dictate the biomechanical behavior of the construct, thus also influence the mechanical strains experienced by the cells. A relatively compliant scaffold would induce larger cellular strains, and subsequent higher tissue prestress, whereas a stiff scaffold could shield the cells from the dynamic loads, yielding a lower prestress. Furthermore, temporal changes in scaffold degradation, ECM accumulation and organization will influence dynamic cues sensed by the cells, and hence the tissue prestress, which then again alters the functioning of the TE construct.

### 4.6. Conclusions

This study investigated how 24 h dynamic loading influences cell traction-induced prestress in 3D microtissues. Toward this end, a setup to culture microtissues was combined with the commercially available Flexcell system to facilitate dynamic culture of the constructs. First, the setup was validated to determine the peak microtissue stretches during the experiment, after which the effect of the applied dynamic microtissue stretch on the generated prestress was quantified. After 24 h, prestress increased significantly compared to static controls. However, after subsequent removal of the dynamic cue, prestress again dropped to levels comparable to static controls. With the addition of a ROCK-inhibitor, the prestress in these microtissues vanished almost completely, confirming that the prestress in these microtissues can be completely attributed to the cellular traction forces. Finally, after removal of the ROCK-inhibitor, prestress magnitudes restored to baseline levels. In conclusions, this study systematically and quantitatively investigated the effect of dynamic loading on cell traction-mediated tissue prestress. The results indicate that intrinsic tissue prestress is a highly controlled parameter, where the actin stress fibers serve as a mechanostat by regulating tissue prestress levels in response to perturbations in the environment. These findings can have important implications for mechanical testing of native cardiovascular tissues, and tissue-engineering therapies.

## Data Accessibility

All data and numerical code have been stored at SURFdrive, a personal cloud storage service for the Dutch education and research community, and are available upon request.

## Data Availability

The datasets generated for this study are available on request to the corresponding author.

## Author Contributions

MvK, NK, SL, and CB conceptualized the idea behind this study. MvK and NK conducted the experiments and numerical simulations and analyzed the data. MvK wrote the manuscript. JF was involved in developing the experimental setup. SL and CB supervised the project. All authors reviewed the manuscript.

### Conflict of Interest Statement

The authors declare that the research was conducted in the absence of any commercial or financial relationships that could be construed as a potential conflict of interest.
