# Supplementary material for: Increased Cell Traction-Induced Prestress in Dynamically Cultured Microtissues
Source: Front Bioeng Biotechnol. 2019 Mar 12;7:41. doi: 10.3389/fbioe.2019.00041 (PMC6422899; doi:10.3389/fbioe.2019.00041)
Supplement: Supplementary file 1 [file Data_Sheet_1.PDF]

Supplementary material for:

M.A.J. van Kelle, N. Khalil, J. Foolen, S. Loerakker and C.V.C. Bouten

Increased cell traction-induced prestress in dynamically cultured microtissues

Determining the micropost spring constant

Spring constants of the microposts were determined using finite element analysis in Abaqus (Dassault Systèmes Simulia Corp., Providence, RI, USA, version 6.14-1). The micropost was modeled as a Neo-Hookean material with a stiffness of 1.72 MPa (Palchesko *et al*), which was fully encastred at the bottom. First, a force was applied to the row of elements just below the large cap of the micropost, which is where the microtissue is attached. Next, the in-plane displacement of the top middle part of the post was determined, which was subsequently used to calculate the micropost spring constant K [N m^{-1}] using $F^i_{post} = Ku_{post}$, with F the force and u_{post} the displacement. The spring constant for the posts used in this study was determined to be 1.22 N m^{-1}.

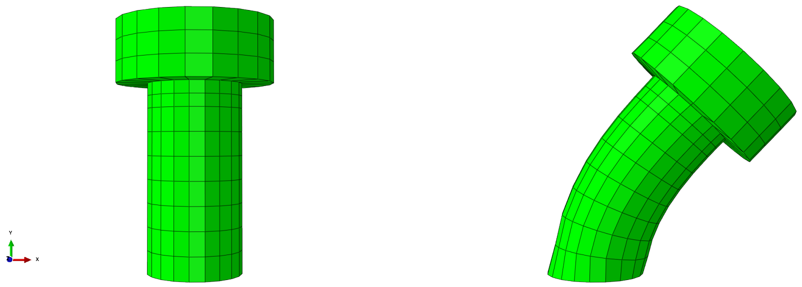

Figure 1: Undeformed micropost (**Left**) and micropost after application of a force to the top part of the post (**Right**)

.

R.N. Palchesko, L. Zhang, Y. Sun and A.W. Feinberg. Development of polydimethylsiloxane substrates with tunable elastic modulus to study cell mechanobiology in muscle and nerve. PLoS One, 2012, 7, e51499

Microtissue Force

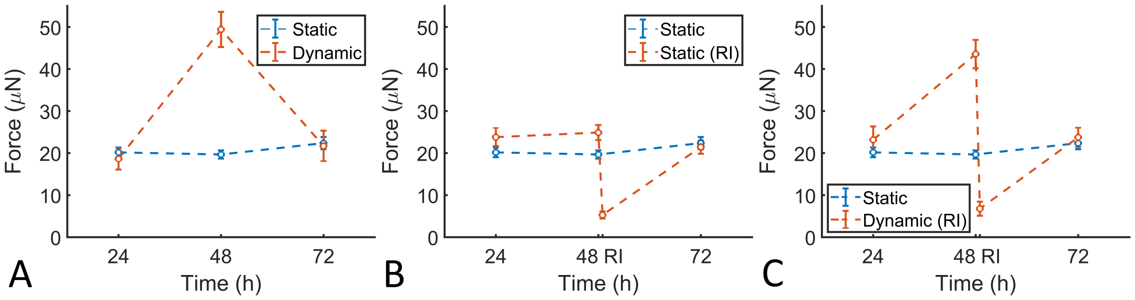

Figure 2: A) Static control group (blue) and dynamically cultured microtissues (red). After application of dynamic mechanical cues, the force increased over two-fold compared to the static controls. Removal of the mechanical load resulted in recovery of microtissue force similar to that of static controls at 72 hours. **B)** Static control group (blue) and ROCK-inhibited (RI, red) microtissues over the course of 72 hours. Initially, force magnitudes in both groups were comparable for up to 48 hours. After addition of the ROCK-inhibitor force magnitudes dropped significantly. Subsequent removal of the inhibitor yielded force recovery comparable to the control group. **C)** Static control group (blue) and dynamically stretched microtissues (red) showed increased force levels after dynamic culture. Adding the ROCK-inhibitor (RI) abated the forces almost completely, yielding comparable prestress magnitudes as ROCK-inhibited static controls in Figure **B**.

Cross-sectional area

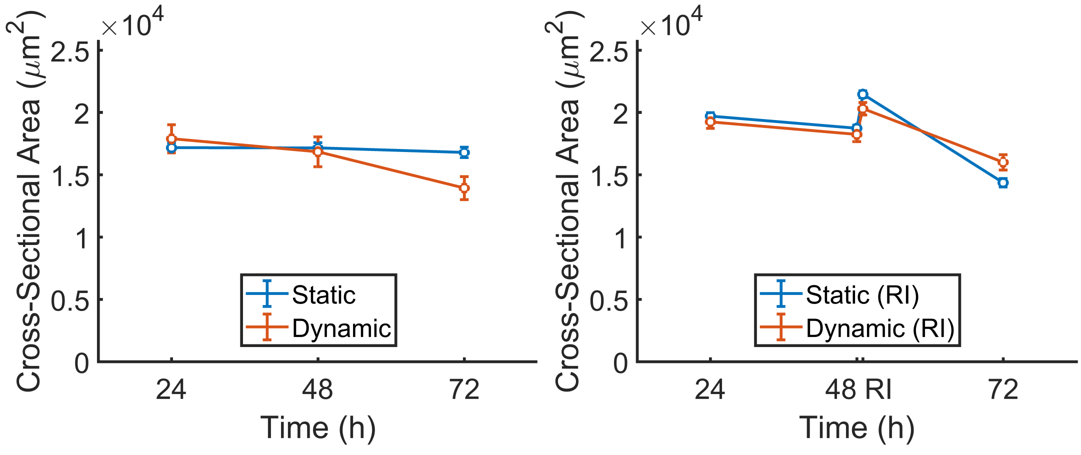

Figure 3: Cross-sectional area (CSA) for static and dynamically cultured microtissues (**Left**) and for the static and dynamically cultured and ROCK-inhibited (RI) microtissues (**Right**). No significant differences at the various time points could be identified.
